# Methaemoglobinaemia Can Complicate Normothermic Machine Perfusion of Human Livers

**DOI:** 10.3389/fsurg.2021.634777

**Published:** 2021-01-28

**Authors:** Samuel J. Tingle, Ibrahim Ibrahim, Emily R. Thompson, Lucy Bates, Ashwin Sivaharan, Yvonne Bury, Rodrigo Figuereido, Colin Wilson

**Affiliations:** ^1^Institute of Transplantation, Freeman Hospital, Newcastle upon Tyne, United Kingdom; ^2^Institute of Cellular Medicine, Newcastle University, Newcastle upon Tyne, United Kingdom; ^3^Department of Cellular Pathology, Royal Victoria Infirmary, Newcastle upon Tyne, United Kingdom

**Keywords:** transplantation, normothermic machine perfusion, blood gas, methaemoglobinaemia, steatosis

## Abstract

**Background:** Although liver normothermic machine perfusion is increasingly used clinically, there are few reports of complications or adverse events. Many centers perform liver NMP to viability test suboptimal grafts, often for prolonged periods. In addition, several researchers are investigating NMP as a drug delivery platform, which usually necessitates prolonged perfusion of otherwise non-viable liver grafts. We describe two instances of methaemoglobinaemia during NMP of suboptimal livers.

**Methods:** The NMP of eight human livers rejected for transplantation is described. Methaemoglobinaeima developed in two; one perfused using generic Medtronic™ perfusion equipment and one using the OrganOx Metra®.

**Results:** The first liver (53 years DBD) developed methaemoglobinaemia (metHb = 2.4%) after 13 h of NMP, increasing to metHb = 19% at 16 h. Another liver (45 years DBD) developed methaemoglobinaemia at 25 h (metHb = 2.8%), which increased to metHb = 28.2% at 38 h. Development of methaemoglobinaemia was associated with large reductions in oxygen delivery and oxygen extraction. Both livers were steatotic and showed several suboptimal features on viability testing. Delivery of methylene blue failed to reverse the methaemoglobinaemia. Compared to a matched cohort of steatotic organs, livers which developed methaemoglobinaemia showed significantly higher levels of hemolysis at 12 h (prior to development of methaemoglobinaemia).

**Conclusions:** Methaemglobinaemia is a complication of NMP of suboptimal liver grafts, not limited to a single machine or perfusion protocol. It can occur within 13 h (a timepoint frequently surpassed when NMP is used clinically) and renders further perfusion futile. Therefore, metHb should be monitored during NMP visually and using blood gas analysis.

## Introduction

Normothermic machine perfusion (NMP) can improve early outcomes in liver transplantation and act as a drug delivery platform (1). Several groups are researching therapeutics which require prolonged NMP in an attempt to utilize suboptimal grafts, which would otherwise be discarded (2–4). In addition, the use of prolonged NMP in the clinical setting is increasing, with human transplants reported after 38 h of NMP (5). However, there is limited experience on the feasibility of performing prolonged perfusion on this heterogenous group of human organs.

Methaemoglobin (metHb) is the oxidized form of hemoglobin, which is incapable of effective oxygen delivery to tissues. Usually metHb is formed at a low level and is salvaged by methaemoglobin reductase within red blood cells (RBC) (6). When this balance is lost metHb accumulates in the blood (or in this case perfusate), termed methaemoglobinaemia (metHb > 2%). Here we describe two instances of methaemoglobinaemia during prolonged liver NMP.

## Methods

Oxygenated perfusion at 37°C is performed at our center using either Organox Metra™ or Medtronic™ perfusion equipment, both of which use a centrifugal pump and Metronic™ oxygenator. Details of perfusion protocols can be found in Table 1. For perfusions with Medtronic™ equipment pressures of 5 and 75 mmHg were used for portal vein and hepatic artery, respectively. Organox Metra™ was used as per the recently published trial (1).

**Table 1:** Comparison of graft details and perfusion protocols for the two livers which developed metHb.

	**Liver 1**	**Liver 2**
**GRAFT DETAILS**		
Donor type	DBD	DBD
Cause of death	Intracranial hemorrhage	Intracranial hemorrhage
Sex	F	M
Age	53	45
Comorbidities	Nil	Nil
Alcohol history	Excessive (7-9 U/day)	Excessive (7-9 U/day)
Steatosis	Severe steatosis^*^ (>50% macrovesicular steatosis on biopsy)	Severe steatosis^*^ (80% total steatosis, 50% macrovesicular steatosis on biopsy)
Liver weight	2.5 kg	2.2 kg
Cold ischaemic time	20 h 33 min	9 h 39 min
**PERFUSION DETAILS**		
Device used	Medtronic™ perfusion equipment	OrganOx Metra™
Oxygen carrier	3 units of human red blood cells	3 units of human red blood cells
Perfusate constituents	Succinylated gelatin (Isoplex) Heparin Sodium bicarbonate Calcium gluconate Cefuroxime	Succinylated gelatin (gelofusine) Heparin Sodium bicarbonate Magnesium sulfate Fluconazole + Meropenem Hydrocortisone Parvolex (Acetylcysteine) Aminoven
Infusions	Heparin Epoprostenol Insulin (6.9 units/h) Cernavit + Synthamin 9	Heparin Epoprostenol Insulin (6.7 units/h) Bile salts

*Similarities between the two cases may be risk factors of methaemoglobin development*.

* *Severe steatosis as deemed by retrieving surgeon. DBD, donation following brainstem death*.

We analyse two perfusions of human discarded livers where methaemoglobinemia occurred. To further elucidate mechanisms we compared these with a matched cohort of 6 additional prolonged perfusions, where metHb levels remained normal throughout.

All of the livers included in this study have been declined for transplant by all UK transplant centers. With the consent of the donor's family, these livers were accepted into approved pre-clinical research projects. Ethical approval for accepting these livers into research projects was granted by the national research ethics commission in the UK, National Research Ethics System (15/SC/0161).

## Results

Our research team has performed NMP of 37 human livers which have been declined for transplant. Two of these livers developed “methaemoglobinaemia,” whilst the remaining 35 maintained metHb levels <2% throughout perfusion. Graft and perfusion details are displayed in Table 1. “Liver 1” was declined on macroscopic appearance prior to initiation of NMP. “Liver 2” was initially accepted for transplantation but subsequently rejected due to steatosis. It was then accepted for research and maintained continuously on NMP.

The following factors were present in both livers (see Table 1): severe steatosis on biopsy, liver weight over 2.2 kg and excessive donor alcohol consumption (7–9 Units/day). Both livers came from donors with elevated weight (83 and 94 kg, respectively) and body mass index (29 and 31 kg/m^2^, respectively). Liver 1 had a prolonged cold ischaemic time (CIT; 20 h 33 min), whilst liver 2 had a CIT deemed acceptable within clinical practice (9 h 39 min). Liver 1 was perfused using generic Medtronic™ perfusion equipment and Liver 2 using the OrganOx Metra® (3). Perfusate constituents and infusions were largely similar.

**Figure 1:**
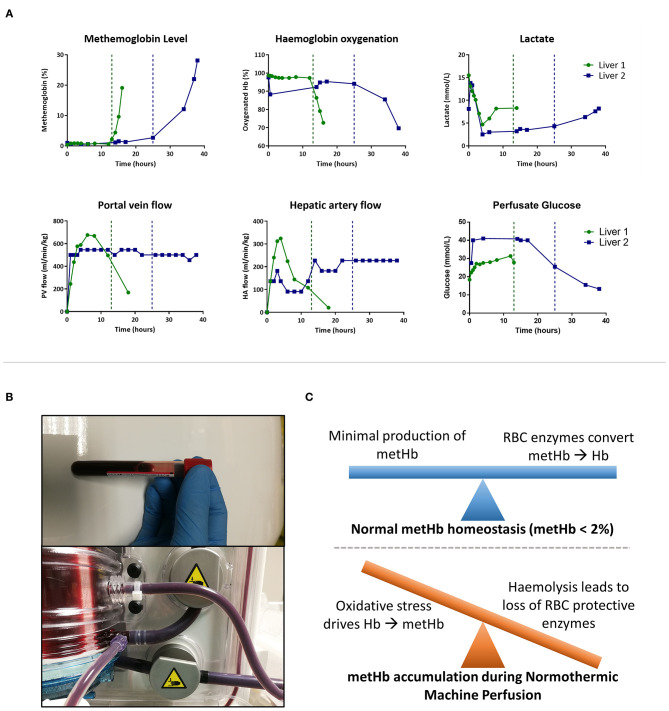
Methaemoglobinaemia (metHb) during normothermic machine perfusion (NMP). **(A)** Perfusate blood gas (from arterial limb) and perfusion flow characteristics throughout NMP. Vertical lines demonstrate when metHb level increases above 2%. **(B)** Perfusate turning chocolate brown then deep purple as metHb level increases. **(C)** Working model of pathogenesis of methaemoglobinemia during NMP.

As shown in Figure 1A, blood gas analysis from the arterial limb of the circuit revealed that Liver 1 developed methaemoglobinaemia (metHb = 2.4%) after 13 h of NMP, increasing to metHb = 19% at 16 h. Liver 2 developed methaemoglobinaemia at 25 h (metHb = 2.8%), which increased to metHb = 28.2% at 38 h. As expected, there was a corresponding fall in oxygenated Hb, as seen in Figure 1A. The development of metHb could easily be seen by a color change in the perfusate from red to chocolate brown and then deep purple (Figure 1B). Methylene blue is often used to treat methaemoglobinaemia clinically. In liver 1 we gave two 50 mg doses of methylene blue, delivered as a bolus into the reservoir at 13 h and 14 h; metHb levels continued to rise despite this intervention.

Satisfactory flow in hepatic artery and portal vein was seen in both livers; at 4 h hepatic artery flow was 810 and 300 mL/min in Liver 1 and 2, respectively, with portal vein flows of 1,470 and 1,200 mL/min, respectively. Weight adjusted flow rates are given in Figure 1A. When metHb developed there was not a reactive increase in arterial or portal venous flow in either liver (Figure 1A). Paired arterial and venous samples were taken in liver 2, allowing calculation of oxygen extraction; this fell from 29.8 ml/min before methaemoglobinaemia developed to 9.8 ml/min (67% reduction) at 38 h. Total oxygen delivery (oxygen available to the liver) was calculated using flow data and oxygen content in arterial limbs of the circuit (calculated with oxygenated hemoglobin percentage, hemoglobin concentration and PaO_2_). Total oxygen delivery to the liver fell significantly following development of methemoglobinemia in both livers. In Liver 1 oxygen delivery fell from 109 ml/min immediately before methaemoglobinaemia development to 45 ml/min (59% reduction) at 16 h. Oxygen delivery fell from 134 ml/min prior to methaemoglobinaemia development, to 50 ml/min (63% reduction) at 38 h in Liver 2.

Despite good flow rates, both livers displayed several features of poor viability during perfusion, prior to development of methaemoglobinaemia. Neither liver maintained a physiological pH; perfusate remained acidotic (pH < 7.20) despite repeated doses of sodium bicarbonate 8.4% (>60 mls in total). ALT at 2 h was 6,628 U/L in liver 1 and 5,896 U/L in Liver 2; the latter being within the range of Watson viability criteria (<6,000 U/L) (7). However, both livers metabolized lactate (Figure 1A); Liver 1 cleared lactate from 15.5 mmol/L initially to 4.7 mmol/L at 4 h and Liver 2 cleared lactate from 13.8 to 2.4 mmol/L at 4 h.

As shown in Figure 1A, glucose metabolism was poor in both livers; in Liver 1 glucose concentration steadily increased from initiation of perfusion to a peak of 31.4 mmol/L at 12 h. Perfusate glucose remained at >40 mmol/L for 18 h in Liver 2, after which it slowly fell to a nadir of 15.5 mmol/L at 34 h. This was despite insulin infusion rates of 6.9 and 6.7 units per hour (Table 2). As glucose concentration remained elevated, neither liver received additional glucose supplementation.

**Table 2:** Graft and perfusion details for severely steatotic livers undergoing prolonged perfusion with or without developing methaemoglobinaemia.

	**Livers developing methaemoglobinaemia (*n* = 2)**	**Livers without methb during 25 h perfusion (*n* = 6)**	***P*-value**
**DONOR TYPE**			
DBD	2	4	1.000
DCD	0	2	
**DONOR SEX**			
Male	1	5	0.464
Female	1	1	
Age (years)	49 ± 5.66	48.67 ± 6.28	0.949
Mean alcohol intake (units/day)	Both 8 U/day	4.50 ± 3.67	N/A
Liver weight (kg)	2.35 ± 0.21	2.55 ± 0.33	0.478
Mean cold ischaemic time (min)	906 ± 462	985 ± 328	0.793
**PERFUSION DEVICE**			
Medtronic™ perfusion equipment	1	6	0.250
OrganOx Metra™	1	0	

*P-values are the result of Fisher's exact tests for categorical variables and t-tests for continuous variables. DBD, donation following brainstem death; DCD, donation following circulatory death*.

Minimal bile was produced by either liver. In liver 2 bile biochemistry was performed at 6 h, which revealed acidotic bile (pH = 7.12) with a glucose concentration lower than perfusate (15.2 vs. 41.0 mmol/L). On the basis of the above, both livers would fail the Cambridge viability criteria at 4 h (1). However, Liver 2 would pass the Birmingham (8) viability criteria at 4 h due to: lactate <2.5, stable and satisfactory flow rates and homogeneous graft perfusion with soft consistency of the parenchyma (1).

Core biopsy samples were analyzed by a Consultant Histopathologist specializing in liver pathology. Liver 1 biopsies taken at 0, 2, 4, and 12 h showed no evidence of bile duct or lobular hepatocyte necrosis. No biopsies were taken after development of methaemoglobinaemia in Liver 1. Liver 2 displayed mild zone 1 necrosis and mild perivenular necrosis at 15 h (metHb <2%). Following development of methaemoglobinaemia, a biopsy at 36 h displayed widespread patchy panzonal necrosis with detachment of biliary epithelium from the basement membrane.

### Comparison With a Matched Cohort

In total, we have performed extended perfusions for ≥24 h in 13 discarded livers, plus one attempted prolonged perfusion which was stopped early due to methaemoglobinaemia (Liver 1). Six livers were declined for reasons other than steatosis and these maintained metHb <2% throughout perfusion. Eight livers were declined due to severe steatosis, including the two livers which developed methaemoglobinaemia.

To further elucidate mechanism, we compared the two livers which developed methaemoglobinaemia within 25 h with a matched cohort of six human livers (also declined for severe steatosis) which underwent NMP for a minimum of 25 h. MetHb level was <2% at all time-points in this matched cohort. Groups were well matched for donor type, age, sex, alcohol intake, liver weight, and cold ischaemic time (Table 2).

**Figure 2:**
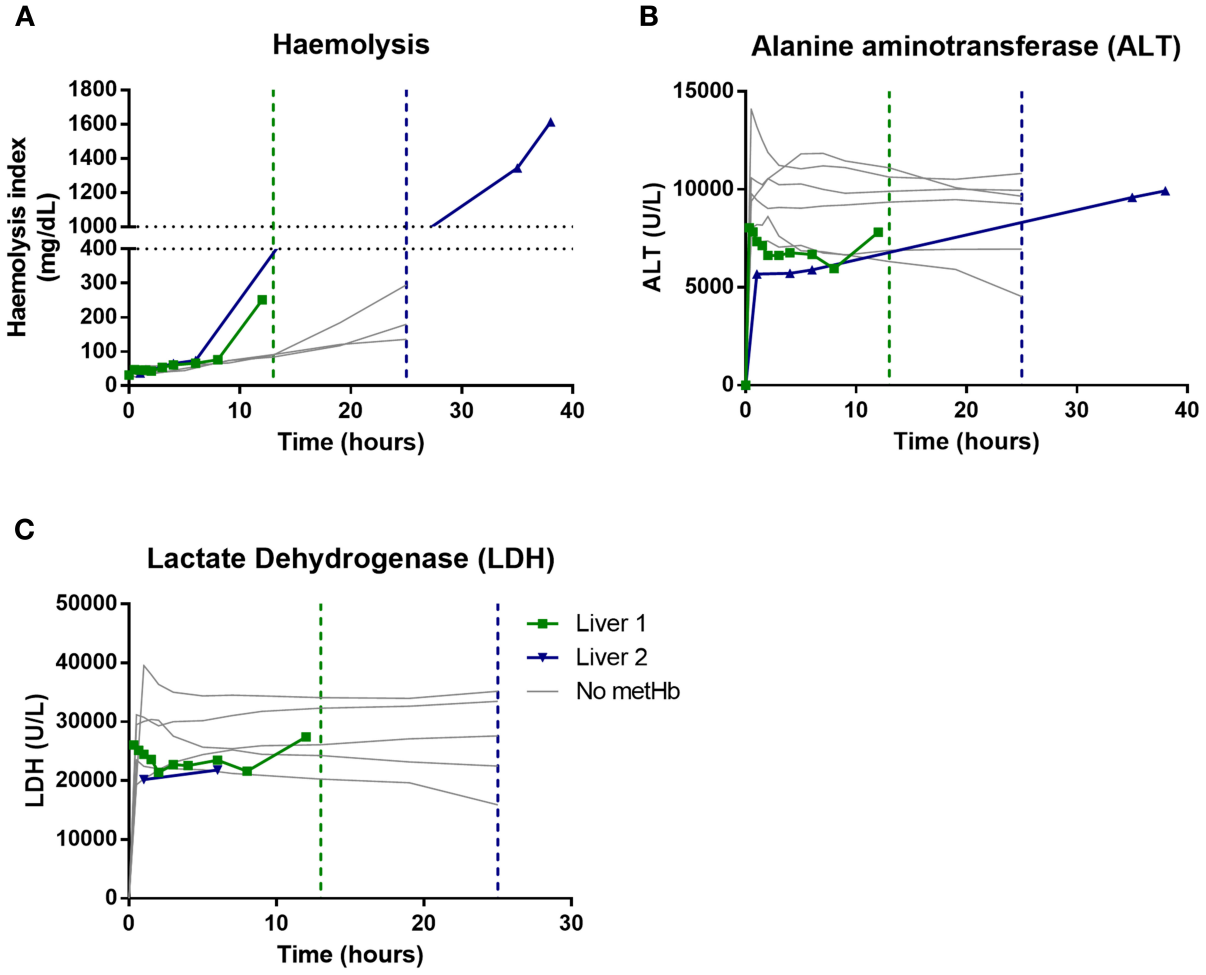
Comparison of severely fatty livers that developed methaemoglobinaemia (Liver 1 and Liver 2 in green and blue, respectively) with those that maintained normal methaemoglobin levels (“No metHb” in gray). Vertical lines demonstrate when metHb level increased above 2%. **(A)** hemolysis index (measured in *n* = 5), **(B)** Alanine aminotransferease (*n* = 8), **(C)** Lactate dehydrogenase (*n* = 7); LDH could not be calculated for later timepoints in liver 2 due to hemolysis.

Hemolysis index increased throughout all perfusions (Figure 2A, data available for *n* = 5 livers). The last hemolysis measurement for liver 1 was at 12 h; mean estimated hemolysis level at 12 h was significantly higher in the livers that subsequently developed methaemoglobinaemia that those that did not (294 vs. 80 mg/dL; *P* = 0.007), as was area under the hemolysis curve up to 12 h (*P* = 0.033). Interestingly, Liver 1 showed a large increase in hemolysis between 8 and 12 h (Figure 2A), which preceded methaemoglobinaemia development at 13 h. ALT and LDH values were high in all of these severely steatotic livers (Figures 2B,C), demonstrating high levels of ischemia reperfusion injury, as expected with severely steatotic organs.

## Discussion

To our knowledge this is the first detailed description of severe methaemoglobinaemia during NMP. We have shown that this complication affects suboptimal liver grafts and is not limited to a single machine or protocol. Methaemoglobinaemia is progressive, readily visible during NMP (Figure 1B), severely impairs oxygen delivery and is resistant to pharmacological reversal with methylene blue, rendering further perfusion futile. Other groups have recorded severe methaemoglobinaemia (>20%) during NMP of suboptimal grafts [Mergental et al. (8), Supplementary Table 1], however this is not reported or discussed in their manuscript or in any further publications (8).

Several research groups are attempting to deliver therapeutics during NMP to improve the quality of otherwise non-viable steatotic liver grafts (3, 4). It is exactly this type of graft and perfusion that is described in the present manuscript; therefore knowledge of this potential complication is vital for the large body of researchers aiming to salvage these grafts.

Observing this complication in two grafts allows us to compare features (Table 1). Overall, perfusion machinery and protocols were very similar; both grafts underwent normothermic perfusion, with a medtronic oxygenator, RBCs used as an oxygen carrier and pressure guided flow into the hepatic artery and portal vein. Perfusate constituents and infusions were also largely similar. Perfusate constituents such as bile salts (taurocholic acid), acetylcysteine and specific antibiotics which were only present in only one protocol (Table 1) are unlikely to be significant contributors to methaemoglobinaemia. The perfusion solutions and constituents used in these livers have all been widely described by other groups (9). Both grafts were severely steatotic, weighed >2.2 kg and came from donors with excessive alcohol consumption. Interestingly, both grafts came from relatively young DBD donors, and CIT was within acceptable limits for Liver 2; these hypothetically protective factors do not prevent methaemoglobinaemia.

In health, metHb levels are kept in balance (Figure 1C) due to low metHb production (low rate of oxidizing events) and salvage of metHb by methaemoglobin reductase which resides alongside hemoglobin molecules inside RBC. Sub-optimal, and especially fatty, liver grafts are known to be sensitive to ischemia reperfusion injury and display significant oxidative stress, which was demonstrated by high ALT and LDH levels (Figures 2B,C). In addition, we have shown that prolonged NMP causes increasing hemolysis (Figure 2A), which will lead to dissociation of hemoglobin molecules from methaemoglobin reductase (2). When comparing two matched cohorts of severely steatotic livers (Table 2), those which progress to methaemoglobinaemia had significantly higher levels of hemolysis at 12 h than those which did not develop methaemoglobinaemia. In Liver 1 a sharp increase in hemolysis at 12 h, preceded methaemoglobinaemia development at 13 h (Figure 1A).

We hypothesize that during NMP of suboptimal grafts a combination of impaired salvage of metHb by methaemoglobin reductase (due to hemolysis) and increased metHb production (severe oxidative stress) shifts the balance toward accumulation of metHb (Figure 1C). Research investigating acellular hemoglobin based oxygen carriers (HBOC) support this hypothesis. HBOC lack methaemoglobin reductase, and when they are used instead of RBC in NMP there are higher levels metHb, which increases steadily during perfusion (6).

There are likely to be a multitude of factors which contribute to both the oxidative stress and RBC hemolysis. The inevitable glucose-6 phosphatase-deficiency which occurs in packed RBC could have a role, as could potential infection of the perfusate. If we experience further episodes of methaemoglobinaemia we will culture the perfusate. However, as broad-spectrum antibiotics are given, we feel that infection is unlikely to play a significant role, especially in the perfusion where methaemoglobinaemia developed at 13 h.

These cases have several implications for clinical practice and research. Some may argue that the findings of this manuscript are not relevant to clinical practice, as methaemoglobinaemia development is only described in suboptimal grafts that may not be transplanted. However, the type of grafts described in this study are the same as those entered into the recently published VITTAL trial (livers initially declined for transplantation in the UK and then accepted into an approved research project) (10). The most common reason (42%) for initial discard in the VITTAL trial was graft steatosis (mirroring the grafts in this study), some of which were transplanted after perfusion. Had liver 2 in this manuscript been instead accepted into the VITTAL trial it would have passed viability criteria at 4 h may therefore have been transplanted (10). This highlights the importance of monitoring metHb level when using NMP for viability testing; especially when recipient factors necessitate prolonged perfusion.

There are also increasing reports of prolonged perfusion in clinical practice, including transplant of organs after 38 h of NMP (5). In the recent randomized trial of liver NMP by Nasralla et al. one quarter of grafts were perfused for more than 11 h 51 min, and the maximum NMP time was over 23 h. It can be inferred that a significant proportion of the cohort were perfused for more than 13 h; the time at which Liver 1 developed methaemoglobinaemia. Similar perfusion times are observed in the VITTAL trial; one quarter of the suboptimal grafts (initially declined for transplantation) were perfused for more than 11 h 45 min (10). To our knowledge measurement of metHb levels is not routine practice, especially for the OrganOx®, where the device provides blood gas analysis (not including metHb). Monitoring for methemoglobinemia both visually and using blood gas analysis should be performed during prolong NMP of suboptimal grafts. This is especially important when HBOC are used, which lack the protective methaemoglobin reductase (6).

In a pre-clinical manuscript Mergental et al. (8) recorded metHb levels of >15% within 2 h of NMP of at least one discarded liver. However, this value is only available in a Supplementary Table and is not reported or discussed anywhere in their manuscript. Although we cannot assess the nature of this graft(s), or how it/they were stored and perfused, this raises concerns that methaemoglobinaemia may complicate even brief periods of NMP.

There is ongoing debate on optimal preservation techniques in liver transplantation. It could be argued that Liver 2 was a viable liver (by the Birmingham viability criteria) at 4 h, and could therefore have been transplanted at this stage. However, due to a multitude of transplant logistics, prolonged preservation is often needed. Perhaps a hypothermic oxygenated perfusion approach, where an oxygen carrier is not required, could be better suited for storage and reconditioning of steatotic grafts akin to those described above (11).

There are also implications for a wide variety of ongoing research projects aiming to recondition these suboptimal grafts with therapies delivered during prolonged perfusion (2–4, 8). Knowledge of this newly reported complication is essential for researchers in this field, as methaemoglobinaemia development rendered further perfusion of these livers futile. Recent research has shown that pulsatile, rather than continuous, perfusion can ameliorate hemolysis which may reduce the risk of methaemoglobinaemia (Figure 1C) (2). In the setting of HBOC, the use of glutathione and vitamin C have been reported to correct or slow metHb accumulation (6). The effect of these therapies during NMP using RBC requires investigation. It is impossible to fully understand NMP methaemoglinaemia based on two cases; reporting of further cases is important to further develop our understanding of risk factors, pathogenesis and potential prophylaxis.

## Conclusion

Methaemglobinaemia is a complication of NMP of suboptimal liver grafts, and is not limited to a single machine or protocol. It can occur within 13 h, a timepoint frequently surpassed when NMP is used clinically (1, 5, 10). Therefore, MetHb should be monitored during NMP both visually and using blood gas analysis, especially when NMP is used for viability testing of suboptimal grafts. We hypothesize that development of methaemoglobinaemia is a combination of hemolytic loss of protective enzymes (methaemoglobin reductase) and the severe oxidative stress which occurs in steatotic livers. Further reporting of cases, and research into preventative strategies are key to increase the number of grafts which can be reconditioned, and minimize graft losses.

## Data Availability Statement

The original contributions presented in the study are included in the article/supplementary material, further inquiries can be directed to the corresponding author/s.

## Ethics Statement

Ethical approval for accepting these livers into research projects was granted by the National Research Ethics Commission in the UK, National Research Ethics System (15/SC/0161).

## Author Contributions

ST, II, ET, LB, AS, RF, and CW: technical perfusion expertise. ST, II, and CW: concept and design. ST: data analysis and drafting article. All authors: data interpretation, critical revision of article, and approval of article.

## Conflict of Interest

The authors declare that the research was conducted in the absence of any commercial or financial relationships that could be construed as a potential conflict of interest.

## Abbreviations

CIT: cold ischaemic time
DBD: donation following brainstem death
HBOC: hemoglobin based oxygen carriers
MetHb: methaemoglobin
NMP: normothermic machine perfusion
RBC: red blood cells.
